# CAR-T cell therapy for autoimmune diseases: current clinical trial landscape and the next wave of development

**DOI:** 10.3389/fimmu.2026.1853170

**Published:** 2026-05-29

**Authors:** Qingyu Chen, Yuxin Zhu, Qianqi Xu, Yemeng He, Yaoyi Wang, Ziyue Wang, Weisong Dong

**Affiliations:** 1 The Second School of Medicine, Wenzhou Medical University, Wenzhou, Zhejiang, China; 2 The First School of Medicine, School of Information and Engineering, Wenzhou Medical University, Wenzhou, Zhejiang, China; 3 Renji College of Wenzhou Medical University, Wenzhou, Zhejiang, China; 4 School of Laboratory Medicine and Life Sciences, Wenzhou Medical University, Wenzhou, Zhejiang, China; 5 School of Nursing and Midwifery, University of Galway, Galway, Ireland; 6 Department of Pathology, The First Affiliated Hospital of Wenzhou Medical University, Wenzhou, China

**Keywords:** autoimmune diseases, BCMA, CAAR, CAR-T, CD19, clinical trials, immune-reset-like remodeling, perspective

## Abstract

Chimeric antigen receptor T-cell (CAR-T) therapy has expanded beyond oncology and is emerging as a promising strategy for autoimmune diseases. Early clinical experience, particularly with CD19-directed CAR-T cells, has shown that deep remission can occur in refractory disorders such as systemic lupus erythematosus, inflammatory myopathies, and systemic sclerosis. These observations are consistent with an immune-reset-like process, although its durability, cellular basis, and disease-specific mechanisms remain incompletely defined. However, the clinical development landscape remains uneven. Based on an April 2026 Trialtrove snapshot, the field is growing rapidly but remains concentrated in a limited number of countries, diseases, and target classes, with most studies in early-phase development. These features suggest that autoimmune CAR-T therapy has moved beyond proof of concept, but has not yet reached a mature, indication-optimized stage of clinical translation. In this Perspective, we argue that the next phase of progress will depend less on increasing trial numbers than on improving biological precision, platform diversity, and trial design. The current pipeline is dominated by CD19-centered programs and diseases in which B-cell depletion appears biologically plausible, but this approach is unlikely to be equally informative across autoimmune disease. Key questions remain regarding remission durability, relapse after B-cell reconstitution, patient selection, toxicity management, and scalability. Looking ahead, major opportunities include plasma cell-directed approaches, dual-target strategies, chimeric autoantigen receptor platforms, tolerance-oriented cell therapies, off-the-shelf products, and *in vivo* engineering. The near-term readout window will be critical in determining whether autoimmune CAR-T therapy becomes broadly deployable or remains limited to selected indications and settings.

## 1 Introduction

The success of CAR-T therapy in hematologic malignancies has reshaped expectations for cellular immunotherapy. More recently, this therapeutic logic has begun to extend into autoimmune disease, where durable remission may be achieved not by broadly suppressing immunity but by selectively removing or reprogramming key pathogenic immune compartments (1, 2). Early clinical reports have generated considerable enthusiasm, especially in severe B-cell-driven diseases, because they suggest that a short-course cellular intervention may induce a degree of disease control that is difficult to achieve with chronic immunosuppression (3–5).

However, the field is now reaching a point where enthusiasm alone is no longer enough. The major question is no longer whether CAR-T can work in autoimmunity, but rather where it is most likely to work, for whom, through which biological mechanisms, and with what degree of long-term safety and scalability. This distinction is important because autoimmune diseases are far more heterogeneous than B-cell malignancies, and the therapeutic objectives are also different. In cancer, the aim is eradication of malignant clones. In autoimmunity, the aim is immune rebalancing, durable remission, organ protection, and ideally treatment-free control without unacceptable toxicity (6).

Against this background, a clinical trial landscape view is useful not simply as a descriptive exercise, but as a way to identify the structure of the field and the decisions that will shape its next phase. Our central argument is that autoimmune CAR-T development has entered a rapid expansion phase, but remains concentrated in a small number of diseases, countries, and target classes, with most programs still positioned in early clinical development. The future of the field will therefore depend on moving from replication of early success toward indication-specific strategy, platform innovation, and better-designed trials.

In this Perspective, we use “immune reset” as a conceptual and hypothesis-generating framework rather than as a fully established mechanistic endpoint. We therefore distinguish between evidence-supported clinical observations, such as drug-free remission and B-cell reconstitution after CD19-directed CAR-T therapy, and mechanistic interpretations that require validation through longitudinal immune profiling.

## 2 Current clinical landscape and developmental signals

Trial records were extracted from Trialtrove using an April 2026 database snapshot. Searches were conducted using terms related to chimeric antigen receptor T-cell therapy and autoimmune or immune-mediated diseases. Eligible records included interventional clinical studies evaluating CAR-T or CAR-T-like cellular therapies in autoimmune indications. Oncology trials, non-CAR cellular therapy studies, duplicate records, and records lacking sufficient information on disease indication or target strategy were excluded. Trials were classified according to start year, country or region, disease indication, target antigen, clinical phase, primary endpoint category, platform type, sponsor or development status, and registry-listed primary completion year. For basket trials including multiple autoimmune indications, disease-level analyses used multi-label expansion, allowing a single trial to contribute to more than one disease category. Classification was based on available Trialtrove fields and manually reviewed trial descriptions.

**Figure 1:**
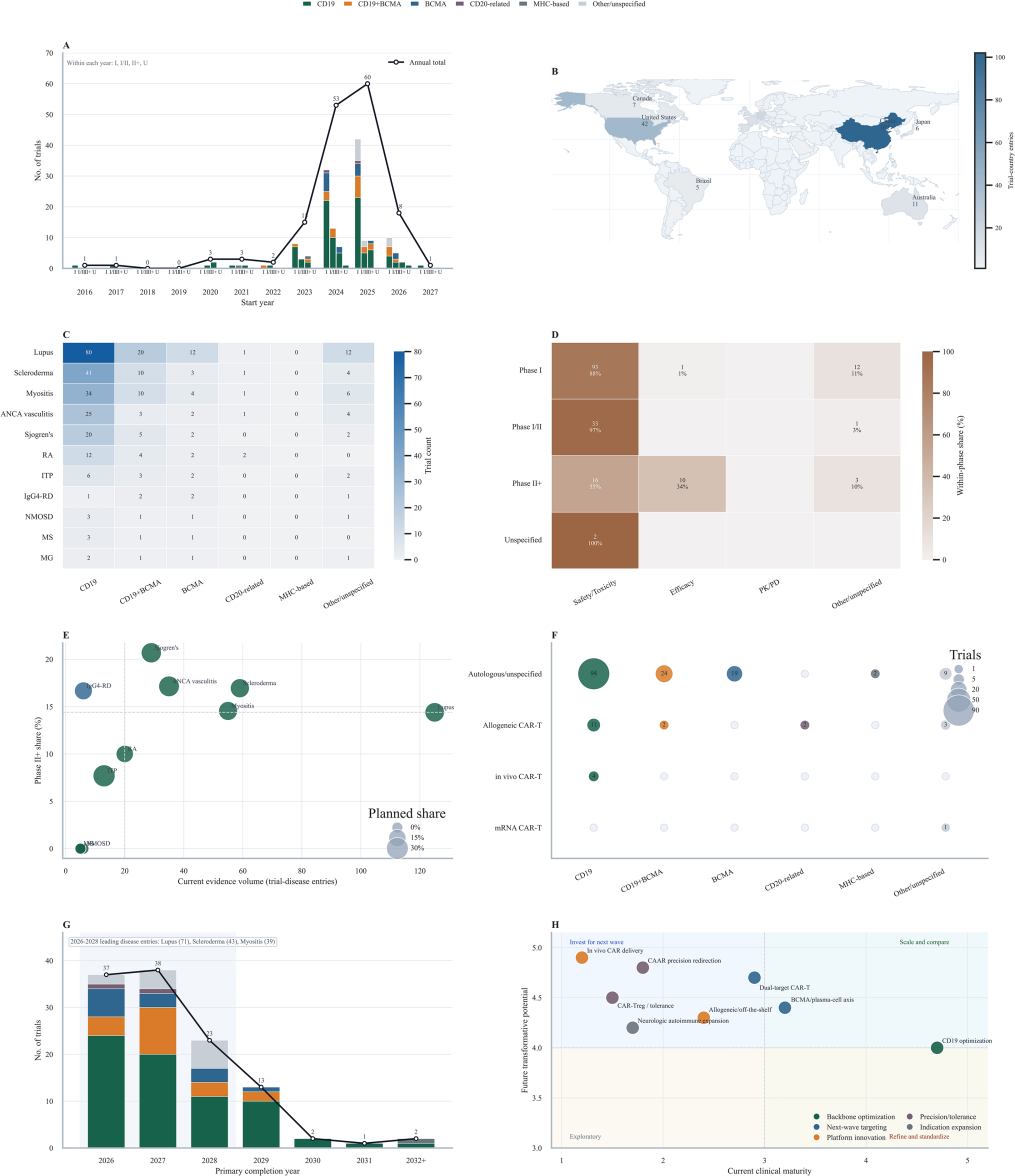
Current landscape, evolution, and future directions of CAR-T clinical development in autoimmune diseases. **(A)** temporal evolution of trials by start year, trial phase, and target class, with the black line indicating the annual total number of trials, including years with zero identified starts. **(B)** global footprint of trial activity; countries are shaded by the number of trial-country entries. **(C)** disease-target architecture across major autoimmune indications. **(D)** phase-endpoint structure, with cells annotated by trial count and within-phase share. **(E)** disease opportunity landscape showing current evidence volume, phase II+ representation, planned-trial share, and dominant target class. **(F)** target-platform diversification across autologous or unspecified, allogeneic, *in vivo*, and mRNA CAR-T approaches. **(G)** projected near-term readout landscape based on registry-listed primary completion year and target class from the April 2026 Trialtrove snapshot; the black line shows the total projected trial readouts per year, and years beyond 2031 were grouped as 2032 +. **(H)** strategic opportunity map highlighting next-wave development priorities according to current clinical maturity and future transformative potential. For panels **(C, E)**, disease-level analyses used multi-label expansion of the disease field; therefore, a basket trial may contribute to more than one disease category. Panel **(B)** uses trial-country entries rather than unique trials. Panel **(H)** is a perspective synthesis informed by the clinical landscape, unmet needs, and platform trajectories rather than a direct registry count. All panels are based on an April 2026 Trialtrove snapshot unless otherwise indicated. Trial classifications were derived from available Trialtrove fields and manually reviewed trial descriptions. Because registry records may be updated over time, trial status and projected primary completion dates should be interpreted as a structured snapshot rather than fixed readout dates.

The present trial landscape shows clear acceleration in activity. Trial starts increased sharply after 2023, with a visible concentration of programs launched in 2024 and 2025, indicating that autoimmune CAR-T is no longer a niche translational effort but an active clinical development area (**Figure 1A**). At the same time, this expansion should not be mistaken for maturity. Most studies remain in Phase I or Phase I/II, and relatively few have advanced into later-stage development. This combination of rapid growth and early-phase concentration is typical of a field that has successfully established proof of concept but is still defining its optimal clinical path.

Geographically, clinical development remains highly concentrated, with trial activity dominated by China, followed by the United States and a smaller group of European countries (**Figure 1B**). This pattern likely reflects differences in manufacturing infrastructure, regulatory flexibility, translational ecosystems, and disease-program prioritization. It also raises an important interpretive issue: evidence generated from a limited set of health-care systems may not fully capture global variation in patient populations, regulatory pathways, manufacturing capacity, or access to advanced cellular therapies. A more globally distributed clinical development landscape will therefore be important for improving generalizability and equitable access (7).

The field is also concentrated by indication and by target. Disease-level analyses show that lupus, scleroderma, inflammatory myopathies, and ANCA-associated vasculitis currently account for a large share of trial-disease entries, whereas several other autoimmune disorders remain relatively underrepresented (**Figures 1C, E**). Likewise, target selection is still dominated by CD19, reflecting the strong translational momentum generated by B-cell depletion and immune-reset-like remodeling as a working concept. This concentration is understandable: diseases with a clear B-cell or plasmablast component offer the most accessible route for early success. Yet it also means that the present clinical landscape should not be overinterpreted as evidence that the same strategy will translate equally well across all autoimmune indications.

## 3 What the current pipeline reveals about field maturity

The structure of current trials provides important information beyond simple counts. Primary endpoints remain heavily weighted toward safety and tolerability, especially in early-phase studies (**Figure 1D**). This pattern confirms that the field is still predominantly exploratory. In practical terms, many ongoing studies are still asking whether a given CAR-T construct can be administered safely in a specific autoimmune setting and whether signals of disease activity improvement justify further development. Fewer studies are yet positioned to answer comparative, registrational, or standard-of-care questions.

This early-stage profile has two implications. First, positive early reports should be interpreted as field-opening signals rather than definitive evidence of broad clinical readiness. Second, the next developmental bottleneck may not be the ability to induce initial remission, but the ability to define which endpoints matter most and how they should be measured. In autoimmune disease, clinically meaningful benefit is multidimensional. It includes disease activity, organ preservation, steroid-sparing, quality of life, and the durability of immune reconstitution. Future trials will need endpoint frameworks that are better matched to these realities than simple short-term safety-led designs.

Platform diversity is also emerging but remains limited in relative terms. Most programs still align with autologous or unspecified CAR-T approaches, whereas allogeneic, *in vivo*, and mRNA-enabled approaches account for a much smaller share of the pipeline (**Figure 1F**). This pattern suggests that the field is currently exploiting the most established engineering route before wider platform diversification. That is a reasonable developmental sequence, but it also means that many of the solutions required for broad implementation, such as manufacturing simplification, turnaround reduction, and improved accessibility, remain incomplete.

## 4 Why current success should not be overgeneralized

A major risk in this field is the assumption that autoimmune CAR-T can advance through simple repetition of the earliest successful disease models. That assumption is unlikely to hold. Autoimmune diseases differ markedly in tissue tropism, pathogenic effector mechanisms, tempo of organ injury, contribution of long-lived plasma cells, and reversibility of damage. A strategy that performs strongly in lupus or myositis may not produce the same depth or durability of benefit in conditions with distinct immune architecture (8, 9).

Target choice is therefore likely to become a defining issue in the next phase of development. CD19 has served as the backbone of the field because it offers a tractable and biologically plausible entry point (10). However, a CD19-centered strategy may leave important compartments insufficiently addressed in some diseases, especially where long-lived plasma cells or other non-CD19-dominant immune populations play a major pathogenic role. The future of the field will likely depend on whether clinical development can move from “B-cell depletion works” to “which immune compartment should be targeted or remodeled in which disease (11)”.

Durability is another unresolved question. One of the most intriguing observations in autoimmune CAR-T is that remission may persist even after B-cell reconstitution. This finding suggests that the therapy may do more than temporarily deplete circulating B cells; it may be consistent with broader immune-network remodeling rather than simple transient B-cell depletion. However, whether this represents a durable and generalizable immune reset remains unresolved and likely varies across diseases, targets, and pathogenic immune compartments. Relapse will probably become a more visible issue as follow-up lengthens and the treated population broadens. A mature field will need mechanistic correlates of durable remission, not only clinical snapshots of early response (3).

Relapse after CAR-T therapy in autoimmune disease may arise through several non-mutually exclusive mechanisms, including incomplete depletion of pathogenic B-cell subsets, persistence of long-lived plasma cells, regeneration of autoreactive memory compartments, tissue-resident immune memory, antigen-independent inflammatory circuits, or insufficient restoration of regulatory immune networks. Distinguishing these mechanisms will be important because they imply different retreatment strategies, including repeat CD19-directed therapy, plasma-cell-directed approaches, dual-target constructs, or maintenance immunomodulation.

**Figure 2:**
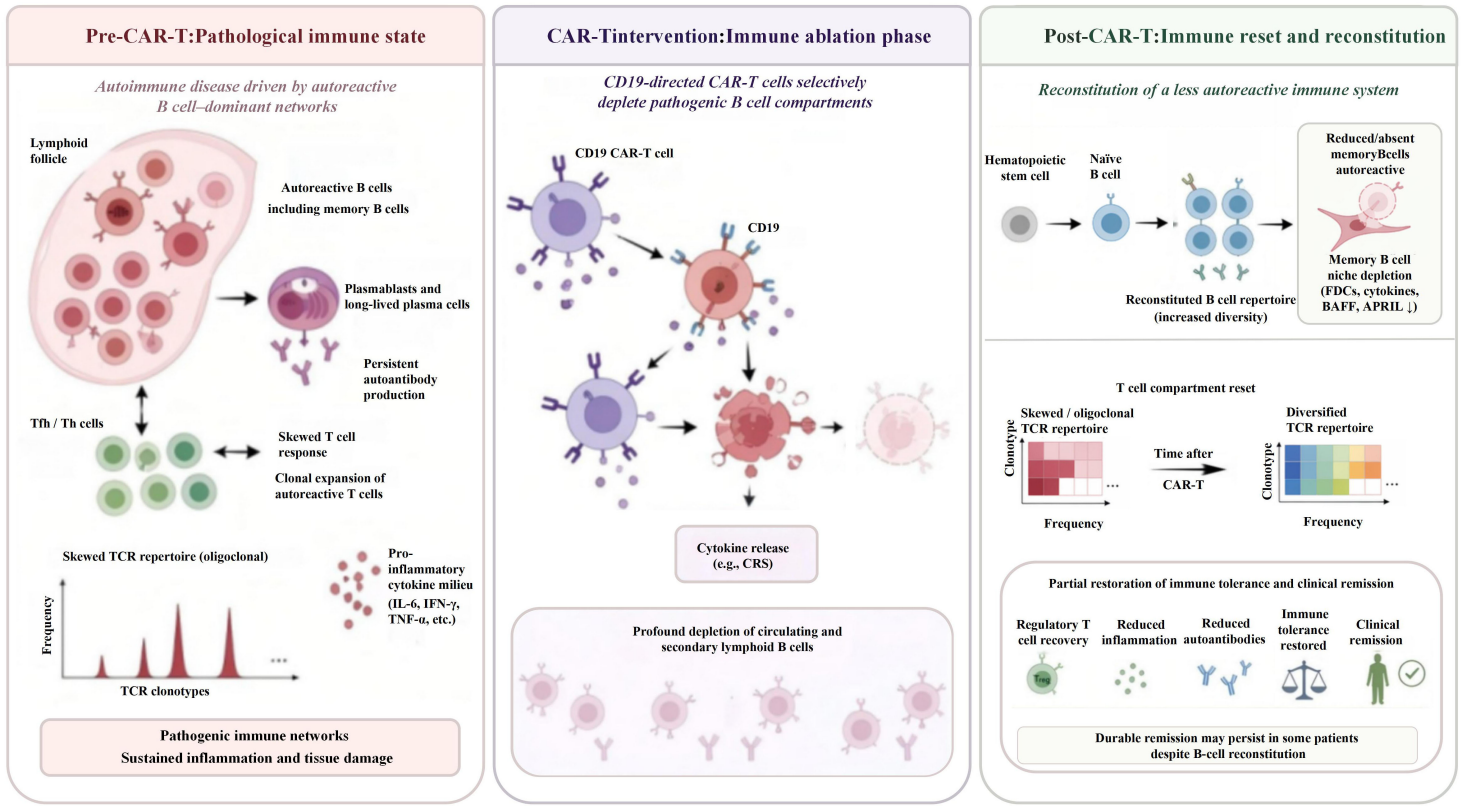
Conceptual working model of immune-reset-like remodeling after CD19-directed CAR-T therapy in autoimmune diseases. **(A)** Before treatment, autoimmune disease is sustained by autoreactive immune networks characterized by pathogenic memory B cells, plasmablast/plasma-cell output, T-cell help, pro-inflammatory cytokine production, and skewed lymphocyte repertoires. **(B)** Following CAR-T infusion, CD19-directed CAR-T cells deplete CD19-expressing B-cell compartments, leading to an acute B-cell depletion phase that may be accompanied by treatment-related toxicities such as cytokine release syndrome (CRS) and immune effector cell-associated neurotoxicity syndrome (ICANS). **(C)** During the reconstitution phase, a less autoreactive immune system may emerge, with repopulation of naive B cells, reduction of autoreactive memory B-cell niches, broader B-cell and T-cell repertoire diversity, and partial restoration of immune tolerance. In some patients, these changes may support durable clinical remission despite B-cell reconstitution, whereas persistence of long-lived plasma cells or other non-CD19-dominant pathogenic compartments may contribute to incomplete remodeling or relapse. This schematic represents a hypothesis-generating framework rather than a fully established biological sequence. The extent to which such immune-reset-like remodeling occurs across different autoimmune diseases requires validation through longitudinal immune profiling.

To better contextualize this unresolved biology, we propose a conceptual working model of immune-reset-like remodeling after CD19-directed CAR-T therapy in autoimmune diseases (**Figure 2**). In this model, remission is not explained solely by transient depletion of circulating B cells, but may reflect broader disruption of pathogenic immune networks, including autoreactive memory B-cell compartments, B-cell/T-cell interactions, inflammatory cytokine circuits, and downstream autoantibody production. During the post-treatment reconstitution phase, repopulation may be biased toward less autoreactive immune states, with expansion of naive B-cell compartments, contraction of autoreactive memory niches, and possible diversification of B-cell and T-cell repertoires. These changes may contribute to persistent clinical remission in some patients despite B-cell reconstitution. However, this process is likely to be disease-dependent and incomplete in some settings, particularly when long-lived plasma cells or other non-CD19-dominant pathogenic compartments remain insufficiently addressed. For this reason, immune reset should be viewed as a biologically plausible, hypothesis-generating framework that requires longitudinal immune profiling and mechanistic follow-up in future trials.

Safety and scalability remain equally central. The toxicity profile of CAR-T, including cytokine release syndrome, neurotoxicity, infection risk, and prolonged immune perturbation, is interpreted differently in autoimmune disease than in aggressive cancer (12). The acceptable risk threshold is often lower, particularly in younger patients or in diseases with chronic but non-immediately fatal courses. In addition, the logistical and economic burden of individualized cellular manufacturing remains a serious constraint. If the field is to expand beyond highly selected refractory cases, it will need platforms that are not only effective but also practical, reproducible, and accessible (13).

Beyond acute toxicities such as CRS and ICANS, several longer-term risks require greater attention in autoimmune populations. Prolonged B-cell aplasia, hypogammaglobulinemia, impaired vaccine responsiveness, recurrent infections, opportunistic infections, and the need for immunoglobulin replacement may substantially affect the risk-benefit balance, particularly in younger patients or in diseases with chronic but non-immediately fatal courses (14). Real-world manufacturing failure, delayed product release, bridging therapy with additional immunosuppression, and variability in cell fitness may also influence feasibility (15). These issues are especially important because the tolerance for severe or prolonged toxicity in autoimmune disease may be lower than in refractory malignancy. Future trials should therefore systematically capture long-term immune reconstitution, infection burden, immunoglobulin use, vaccination responses, hospitalization, and patient-reported recovery.

Economic accessibility also remains unresolved. Even if efficacy is confirmed, specialized manufacturing, lymphodepletion, inpatient or closely monitored administration, toxicity management, long-term follow-up, potential immunoglobulin replacement, reimbursement uncertainty, cost, and infrastructure requirements may limit equitable access across health-care systems.

## 5 Future directions: from expansion to strategy

The next several years are likely to be decisive for the field. Registry-based primary completion timelines indicate a dense near-term readout window, especially across 2026 to 2028 (**Figure 1G**). This matters because the key issue is no longer whether isolated dramatic responses can occur, but whether signals begin to converge across diseases, targets, and development settings. If multiple readouts confirm durable and reproducible benefit, the field could move rapidly toward more standardized development pathways. If results are more variable, the lesson may be that autoimmune CAR-T requires sharper biological and clinical segmentation than is currently being applied.

In our view, the next wave of progress will come from strategic differentiation rather than simple numeric growth. Several directions appear especially important (**Figure 1H**). First, plasma-cell-directed and BCMA-oriented strategies may be biologically attractive in diseases where autoantibody production and long-lived plasma-cell biology are central, but their comparative advantage over CD19-directed approaches remains unproven in autoimmune indications and will require disease-specific validation. Second, dual-target approaches may help address immune redundancy or incomplete single-compartment depletion, although added toxicity, manufacturing complexity, and target-selection uncertainty require careful evaluation. Third, CAAR-based approaches may offer greater antigen specificity in selected autoantibody-mediated diseases, but their applicability is likely to be highly indication-dependent and requires well-defined pathogenic antigens (16). Fourth, tolerance-oriented cellular therapies, including CAR-Treg concepts, are conceptually promising in settings where immune restoration rather than immune ablation is the desired goal, but their durability, specificity, and clinical efficacy remain to be established (17).

Platform innovation may be just as important as target innovation. Allogeneic or off-the-shelf products could reduce manufacturing time and broaden access in principle, but their real-world value will depend on persistence, host rejection, repeat dosing feasibility, safety, regulatory complexity, and cost. Similarly, *in vivo* engineering approaches may eventually improve scalability, but they remain clinically immature in autoimmune indications and require careful validation of delivery, specificity, durability, and safety. These innovations may prove important if autoimmune CAR-T is to become a realistic therapeutic platform rather than a specialized rescue strategy, but they should not yet be interpreted as established solutions to scalability.

## 6 What better trials should look like

The field now needs better trials, not simply more trials. Future studies should define patient selection more precisely, including disease stage, immunologic phenotype, prior treatment exposure, and extent of organ involvement (18). They should also move beyond early safety-focused outcome structures and capture not only remission, glucocorticoid sparing, organ-specific recovery, quality of life, and long-term immune reconstitution, but also serious infection burden, duration of cytopenia, immunoglobulin trajectories, immunoglobulin replacement, vaccine responsiveness, hospitalization, manufacturing success, time from leukapheresis to infusion, reasons for production failure, use of bridging therapy, outcomes of enrolled-but-not-infused patients, and retreatment outcomes.

Longitudinal immune monitoring should become a core component of future autoimmune CAR-T trials. Relevant measures may include B-cell subset reconstitution, naive-to-memory B-cell ratios, plasmablast and plasma-cell dynamics, autoantibody trajectories, T-cell repertoire diversity, cytokine profiles, regulatory T-cell recovery, vaccine responsiveness, immunoglobulin levels, and tissue- or organ-specific inflammatory biomarkers. These analyses would help distinguish transient lymphocyte depletion from durable immune-network remodeling and may identify predictors of relapse, incomplete response, or sustained drug-free remission.

Standardization will also be essential. If definitions of remission, follow-up duration, biomarker reporting, safety endpoints, and manufacturing outcomes remain inconsistent, cross-trial interpretation will be difficult and progress toward indication-specific development will be slowed. Comparative thinking should begin to enter the field through platform comparisons, disease-specific development strategies, or, ultimately, head-to-head positioning against high-intensity biologics or transplant-adjacent approaches in selected settings.

## 7 Conclusion

CAR-T therapy for autoimmune diseases has moved decisively beyond proof of concept. The current clinical landscape shows a field that is expanding rapidly, but one that remains geographically concentrated, biologically centered on a limited number of targets, and largely confined to early-phase development. These features do not diminish the significance of current progress; rather, they clarify what must happen next.

The future of autoimmune CAR-T will not be determined simply by whether more trials are initiated. It will be determined by whether the field can identify the right diseases, the right immune compartments, the right engineering platforms, and the right trial designs to translate early promise into durable, scalable, and indication-specific benefit. This transition will require clearer separation between evidence-supported clinical observations and hypothesis-driven mechanisms, together with direct validation of long-term safety, manufacturing feasibility, and economic accessibility. In that sense, the most important transition ahead is not from few trials to many trials, but from enthusiasm-driven expansion to biologically and clinically disciplined development.

## Data Availability

Publicly available datasets were analyzed in this study. This data can be found here: https://www.citeline.com/en/products-services/clinical/trialtrove.
